# Cpd-42 protects against calcium oxalate nephrocalcinosis-induced renal injury and inflammation by targeting RIPK3-mediated necroptosis

**DOI:** 10.3389/fphar.2022.1041117

**Published:** 2022-11-03

**Authors:** Bingbing Hou, Mingming Liu, Yang Chen, Weijian Ni, Xiaoguo Suo, Yuexian Xu, Qiushi He, Xiaoming Meng, Zongyao Hao

**Affiliations:** ^1^ Department of Urology, The First Affiliated Hospital of Anhui Medical University, Hefei, China; ^2^ Institute of Urology, Anhui Medical University, Hefei, China; ^3^ Anhui Province Key Laboratory of Genitourinary Diseases, Anhui Medical University, Hefei, China; ^4^ The Key Laboratory of Anti-inflammatory of Immune Medicines, Inflammation and Immune Mediated Diseases Laboratory of Anhui Province, Anhui Institute of Innovative Drugs, School of Pharmacy, Ministry of Education, Anhui Medical University, Hefei, China

**Keywords:** calcium oxalate, nephrocalcinosis, inflammation, necroptosis, ripk3, compound 42

## Abstract

Calcium oxalate (CaOx) crystals, as the predominant component of human kidney stones, can trigger excessive cell death and inflammation of renal tubular epithelial cells, involved in the pathogenesis of nephrocalcinosis. Necroptosis mediated by receptor-interacting protein kinase 3 (RIPK3) serves a critical role in the cytotoxicity of CaOx crystals. Here, we assessed the therapeutic potential of a novel RIPK3 inhibitor, compound 42 (Cpd-42), for CaOx nephrocalcinosis by comparison with dabrafenib, a classic RIPK3 inhibitor. Our results demonstrated that Cpd-42 pretreatment attenuated CaOx crystals-induced renal tubular epithelial cell (TEC) injury by inhibiting necroptosis and inflammation *in vitro* and *in vivo*. Furthermore, in an established mouse model of CaOx nephrocalcinosis, Cpd-42 also reduced renal injury while improving the impaired kidney function and intrarenal crystal deposition. Consistent with this finding, Cpd-42 was confirmed to exhibit superior inhibition of necroptosis and protection against renal TEC injury compared to the classic RIPK3 inhibitor dabrafenib *in vitro* and *in vivo*. Mechanistically, RIPK3 knockout (KO) tubular epithelial cells pretreated with Cpd-42 did not show further enhancement of the protective effect on crystals-induced cell injury and inflammation. We confirmed that Cpd-42 exerted protective effects by specifically targeting and inhibiting RIPK3-mediated necroptosis to block the formation of the RIPK1-RIPK3 necrosome. Taken together, targeted inhibition of RIPK3-mediated necroptosis with Cpd-42 may provide a potential therapeutic approach for CaOx nephrocalcinosis.

## 1 Introduction

Kidney stones affects approximately 1–20% of the world’s population. Calcium oxalate (CaOx) crystals, as the predominant component of kidney stones in humans, are accounting for more than 80% (Turk et al., 2016). Various diseases with excessive oxalate excretion (primary hyperoxaluria, acquired gastrointestinal-related hyperoxaluria, consumption of oxalate-rich foods, excessive intake of vitamin C and ethylene glycol, etc) can cause the precipitation of CaOx crystals in renal tubules lumen, which trigger excessive cell death and inflammation resulting in injury to the tubular epithelial cell (TEC) and then in turn promote crystal adhesion to the injured TECs and aggravate crystal deposition in the kidney to form nephrocalcinosis, which also contributes to acute and chronic kidney diseases and kidney stones (Liu et al., 2019; Khan et al., 2021; Demoulin et al., 2022). Oxalate-associated kidney diseases are more likely to occur in patients with pre-existing chronic kidney diseases (Shee and Stoller, 2022). Moreover, kidney injury induced by CaOx crystals is often irreversible and can even progress to end-stage renal disease (Ix, 2019; Waikar et al., 2019; Shee and Stoller, 2022). Nasr et al. (2008) reported that only 27% of patients experienced functional recovery. In this regard, it is important and urgent to develop potentially effective drugs for the treatment of CaOx nephrocalcinosis.

Intrarenal CaOx crystals often triggers excessive intrarenal inflammation (Liu et al., 2019; Khan et al., 2021) and renal TECs necroptosis (Mulay et al., 2016). Necroptosis is a caspases 8-independent of cell death mediated by tumour necrosis factor 1 (TNFR1)/receptor-interacting protein kinase (RIPK)/Mixed lineage kinase domain-like (MLKL) signalling (Dannappel et al., 2014), which is associated with tumours (Mrkvova et al., 2021; Brown, 2022), autoimmune diseases (Tonnus et al., 2021), neurodegenerative diseases (Yuan et al., 2019; Mrkvova et al., 2021; Yu et al., 2021), metabolic diseases (Afonso et al., 2021; Brown, 2022), etc. Several studies have demonstrated that necroptosis also served an essential role in the injury induced in multiple models of kidney disease, like renal ischaemia/reperfusion injury, cisplatin-induced and sepsis-induced acute kidney injury, including crystalline nephropathy, and knockout or pharmacological blockade of necroptosis-mediated critical kinases such as MLKL, RIPK3, RIPK1 and TNFR1 attenuates renal injury (Linkermann et al., 2012; Mulay et al., 2016; Meng et al., 2018; Landau et al., 2019; Priante et al., 2019; Marquez-Exposito et al., 2021).

RIPK3 is a key regulatory protein that mediates the necroptosis signalling pathway, and the deletion of RIPK3 can completely block the occurrence of necroptosis (Newton et al., 2014; Zhang et al., 2020). With further research, it has been found that RIPK3 is also involved in the activation of inflammasomes and the regulation of various inflammatory signals (Lawlor et al., 2015; Evans and Coyne, 2019; Afonso et al., 2021). Dabrafenib, the unique inhibitor of ATP competitive RIPK3 type I (Rheault et al., 2013; Li et al., 2014), has been reported to attenuate poisonous epidermal necrolysis (Kim et al., 2015), hepatocyte necrosis induced by acetaminophen (Li et al., 2014), brain injury induced by ischaemia (Cruz et al., 2018) and the cytotoxicity of various crystals (Mulay et al., 2016). However, dabrafenib is a nonspecific inhibitor of RIPK3 kinase and is widely used against tumours as a threonine/serine kinase BRAF^V600E^ inhibitor, which may come with potentially redundant side effects. In the present study, we synthesized a novel RIPK3 inhibitor, compound 42 (Cpd-42), and tested its anti-necroptotic effect in CaOx crystal-induced cell injury, while its attenuating effects on inflammation was also determined *in vitro* and *in vivo* compared with those of dabrafenib. Notably, the effects of Cpd-42 were assessed by administration protocols at different doses and times on CaOx nephrocalcinosis mice.

## 2 Materials and methods

### 2.1 Chemicals and reagents

Cpd-42, a derivative of the pan-Raf inhibitor TAK-632 (Degterev et al., 2005), was discovered by Zhang H et al. (2019) by optimizing TAK-632 to enhance the inhibitory activity of RIPK3 while exhibiting improved oral bioavailability in rats. Cpd-42 used in this study was synthesized following the procedure described in the literature, and ^1^H nuclear magnetic resonance and high-resolution mass spectrometry (Fig. S1) were employed to confirm the chemical structure of the synthesized Cpd-42, which is consistent with the previous literature (Zhang et al., 2019). The purity of Cpd-42 was determined to be above 95% by high-performance liquid chromatography.

Glyoxylate and calcium oxalate monohydrate (COM) were obtained from Sigma–Aldrich (Sigma, G10601 and C0350000), and dabrafenib was obtained from MedChemExpress Biotechnology (MCE, HY-14660A). The antibodies involved in the present study are as follows: anti-KIM1 (ab78494, IF: 1:200, WB: 1:1000), anti-RIPK3 (ab226297, WB: 1:1000) and anti-phospho-RIPK3 (ab195117, WB: 1:800) from Abcam; anti-p65 (CST8242, WB: 1:1000), anti-phospho-p65 (CST3033, WB: 1:1000), anti-MLKL (CST14993, WB: 1:1000) and anti-phospho-MLKL (CST33733, WB: 1:1000) from Cell Signaling Technology; anti-β-actin (bsm-33036 M, WB: 1:800) and anti-TNF-α (bs-10802R, IHC: 1:200) from Bioss; anti-RIPK1 (sc-7881, IP: 1:500) from Santa Cruz Biotechnology; and F4/80 (GB11027, IHC: 1:500) from Servicebio; Blood urea nitrogen (BUN) and creatinine (Cr) assay kits from Nanjing Jiancheng Bioengineering Institute; Cell Counting Kit-8 (CCK-8) from Topscience; Periodic acid-Schiff (PAS) staining kit from Solarbio.

### 2.2 Cell culture

The human kidney TEC (HK-2) line was obtained from the Institute of Basic Medical Sciences, Chinese Academy of Medical Sciences and routinely cultured in 10% foetal bovine serum (FBS) and DMEM/F12 mixture medium in a humidified atmosphere of 5% CO_2_ and 95% air. HK-2 cells were pretreated with Cpd-42 (0.05, 0.1 and 0.2 μM) or dabrafenib (10 μM) for 0.5 h before being stimulated with COM (1,000 mg ml^−1^) for 24 h after starvation in 0.5% FBS and DMEM/F12 mixture medium with 0.5% FBS overnight (Mulay et al., 2016).

### 2.3 Generation of RIPK3 knockout cells

We generated a RIPK3 knockout mouse kidney TEC (MTEC) line using the CRISPR‒Cas9 system. Lentiviral vectors with Cas9 and exon 3 targeting murine-derived RIPK3 were provided by Shanghai Genechem. In brief, MTECs were infected using green fluorescent protein (GFP)-labelled lentivirus, and then, infected cells were sorted according to GFP expression using flow cytometry. MTECs with GFP alone were inoculated in 96-well plates and cultured for 7–10 days. Validation of RIPK3 knockdown in isolated monoclonal cells was conducted by PCR and DNA sequencing.

### 2.4 Cell viability determination

CCK-8 was employed for the determination of cell viability. Briefly, HK2 cells were inoculated in 96-well plates and pretreated with various concentrations (0.05–20.0 μM) of Cpd-42 or dabrafenib with or without COM (1,000 mgml^−1^) stimulation for 24 h. Then 10 μL of CCK-8 solution was added to each well and incubated for 60–90 min. The optical density at 492 nm was analysed (Multiskan MK3, Thermo, United States) following the instructions of the manufacturer. A Calcein/Propidium Iodide (PI) Cell Viability/Cytotoxicity Assay Kit was employed to distinguish dead and live cells. Briefly, cells were incubated with Calcein AM/PI assay working solution for 0.5 h at 37°C and observed under a microscope (Olympus IX83, Japan). Live cells were stained by calcein AM and presented with green fluorescence, while dead cells were stained by PI and presented with red fluorescence.

### 2.5 Animal experiments

Six-to eight-week-old male C57BL/6J mice were procured from the Laboratory Animal Center of Anhui Medical University. All animal experiments were performed at Anhui Medical University. The Animal Experimentation Ethics Committee of Anhui Medical University approved all animal experiments (No: LLSC20221251). For establishment of the CaOx nephrocalcinosis mouse models, mice received intraperitoneal injections of glyoxylate at 100 mg/kg every day, and all mice were sacrificed after 7 days of injection. For Cpd-42 treatment, mice received intragastric administration of either Cpd-42 (6, 12 and 24 mg/kg) or dabrafenib (100 mg/kg) 12 h prior to glyoxylate administration, followed by intragastric administration once daily. The kidney and blood were harvested at sacrifice for further experiments. The obtained blood was used for Cr and BUN detection following the instructions of the manufacturer, and kidney tissues were routinely subjected to paraffin embedding and molecular studies.

### 2.6 Histomorphology and immunohistochemistry

Mouse paraffin-embedded kidney sections were prepared *via* standard histopathological techniques, including fixation with 4% paraformaldehyde, dehydration, waxing, embedding and sectioning (4 μm), and stained with haematoxylin and eosin (H&E). For IHC, kidney sections were subjected to antigen repair, blocked for endogenous peroxidase activity and incubated with anti-F4/80, anti-TNF-α and anti-KIM1 overnight at 4°C, followed by incubation of secondary antibodies for 30 min at room temperature and dyeing with DAB. All images were acquired by a microscope (Olympus IX83, Japan).

### 2.7 Tubular injury determination

For evaluation of tubular injury, PAS staining was conducted with a PAS staining kit following routine protocols. The tubular injury score was determined by three experienced pathologists based on graded brush border loss, tubular dilation and atrophy, and intraluminal cast formation. The rating criteria of tubular lesions were as follows (Liu et al., 2019): 0 (normal), 1 (≤10%), 2 (11–25%), 3 (26–50%), 4 (51–75%), and 5 (≥76%).

### 2.8 Immunofluorescence staining

Cells were seeded on glass slides, fixed with paraformaldehyde and then incubated with anti-KIM1 at 4°C overnight. After being washed with PBS, the cells were incubated with the appropriate secondary antibody for 2 h at 37°C and then stained with DAPI. All images were acquired by fluorescence microscopy (Olympus IX83, Japan).

### 2.9 Detection of renal CaOx crystals

Intraperitoneally injected glyoxalate was metabolized in the liver to oxalate, which was excreted *via* the kidneys to form CaOx crystals. Polarized light optical micrographs (Zeiss, Oberkochen, Germany) of H&E-stained sections were applied to visualize renal CaOx crystal deposition. The percentage of CaOx crystal deposition in each kidney section was determined using ImageJ software.

### 2.10 Western blot analysis

Protein from cultured cells and renal tissues was extracted by standard protocols and subjected to Western blot analysis according to a previous description (Wang et al., 2019). In brief, the proteins were electrophoresed on 10% SDS/PAGE and then transferred to a nitrocellulose membrane. After blocking of nonspecific binding, the membranes were incubated with anti-β-actin, anti-KIM1, anti-phospho-RIPK3, anti-RIPK3, anti-phospho-MLKL, anti-MLKL, anti-p65, and anti-phospho-p65 overnight at 4°C, followed by incubation with the secondary antibody for 90 min at 37°C. The protein bands were acquired by a Licor/Odyssey infrared imaging system (Li-COR Biosciences, United States). Quantitative analysis was conducted using ImageJ software by measuring the greyscale value of the bands.

### 2.11 RNA extraction and real-time PCR

Total RNA from cultured HK2 cells and kidney tissues was isolated using TRIzol following the manufacturer’s instructions. Quantification of RNA concentration was determined using a NanoDrop 2000 Spectrophotometer. Real-time quantitative PCR was carried out using Bio-Rad iQ SYBR Green Supermix with Opticon two in a CFX96 real-time RT‒PCR detection system (Bio-Rad, United States). The primers used in the present study are shown in Table 1.

**TABLE 1:** Primers sequences used in this study.

Terms	Forward primer (5′–3′)	Reverse primer (5′–3′)
Mice		
KIM1	CTG​CAG​GGA​GCA​ATA​AGG​AG	TCC​AAA​GGC​CAT​CTG​AAG​AC
TNF-α	CAT​CTT​CTC​AAA​ATT​CGA​GTG​ACA​A	TGG​GAG​TAG​ACA​AGG​TAC​AAC​CC
MCP-1	CTT​CTG​GGC​CTG​CTG​TTC​A	CCA​GCC​TAC​TCA​TTG​GGA​TCA
β-actin	CAT​TGC​TGA​CAG​GAT​GCA​GAA	ATG​GTG​CTA​GGA​GCC​AGA​GC
Human		
TNF-α	CAT​CTT​CTC​AAA​ATT​CGA​GTG​ACA​A	TGG​GAG​TAG​ACA​AGG​TAC​AAC​CC
MCP-1	CAG​CCA​GAT​GCA​ATC​AAT​GCC	TGG​AAT​CCT​GAA​CCC​ACT​TCT
β-actin	CGCCGCCAGCTCACCATG	CACGATGGAGGGGAAGAC

### 2.12 Cellular thermal shift assay

CETSA was performed following a previously described protocol (Wang et al., 2019; Wang et al., 2022). Briefly, HK2 cells were processed with Cpd-42 or vector control for 0.5 h and then stimulated with COM. Protein extracts were quantified to obtain the same concentration and evenly distributed in six tubes. The samples were denatured on a CFX96 PCR instrument (Bio-Rad) at different temperatures for 8 min and then freeze‒thawed using liquid nitrogen three times. Finally, supernatants obtained from centrifuged samples were subjected to Western blot analysis.

### 2.13 Coimmunoprecipitation

For Co-IP analysis, the protocol was performed by following a previous description (Wang et al., 2019; Wang et al., 2022). Cells were lysed in precooled RIPA buffer. Protein extracts were precipitated by incubation overnight at 4°C with 1 μg anti-RIPK1 and protein A/G-agarose beads (Santa Cruz, United States). The RIPK1-agarose bead complexes were washed with precooled RIPA lysis buffer containing PMSF three times. The RIPK1-binding proteins in the beads were extracted with boiled SDS and further analysed by Western blots with anti-RIPK3.

### 2.14 Statistical analyses

The quantitative data acquired from this work are shown as the mean ± SEM. One-way ANOVA or independent samples t test followed by the Newman–Keuls post hoc test were employed to determine the differences in means between the groups. SPSS^®^ 23.0 was employed to perform statistical analysis. *p* values < 0.05 were considered to be statistically significant.

## 3 Results

### 3.1 Cpd42 attenuated COM crystal-induced HK2 cell injury

**FIGURE 1:**
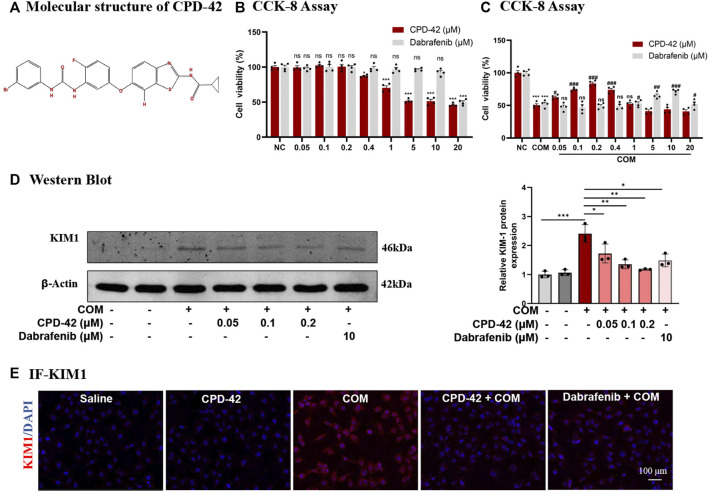
Compound 42 (Cpd-42) prevents calcium oxalate monohydrate (COM) crystal-induced cell injury. **(A)** Molecular structure of Cpd-42. **(B)** The cytotoxicity of Cpd-42 and dabrafenib at different concentrations on HK2 cells was determined by CCK-8 assays. **(C)** Pretreatment with Cpd-42 and dabrafenib restored cell viability in the COM crystal-exposed HK2 cells. **(D)** Protein expression of KIM1 was determined by Western blots of the COM crystal-stimulated HK-2 cells with or without Cpd-42. **(E)** Immunofluorescence of KIM1 in HK-2 cells. Data are shown as the mean ± S.E.M. of at least 3 biological replicates. *p < 0.05, **p < 0.01, ***p < 0.001 compared with the medium control; ###*p* < 0.001 compared with the COM crystal-exposed group.

Cpd-42 was successfully synthesized, and Figure 1A showed its molecular structure. The cytotoxicity of Cpd-42 on HK2 was first assessed, and the results of the CCK-8 assay showed that Cpd-42 at concentrations below 0.4 μM had almost no effect on the viability of HK2 cells (Figure 1B). Next, we further investigated the effect of Cpd-42 on COM crystal-induced HK2 cell injury. CCK-8 assay results indicated that COM crystal exposure resulted in a significant decrease in HK2 cell viability, but the decrease in cell viability was improved by Cpd-42 (Figure 1C), with concentration-dependent effects at 0.05, 0.1, and 0.2 μM, and the improved cell viability of Cpd-42 at the optimal concentration (0.2 μM) was stronger than that of dabrafenib at the optimal concentration (10 μM) (Mulay et al., 2016), a classical RIPK3 inhibitor (Figure 1C). Furthermore, we evaluated the expression of KIM1, which is a marker of injured renal tubules, and Western blot results demonstrated that COM crystals upregulated KIM1 expression; however, Cpd-42 (0.2 μM) significantly inhibited the expression of KIM and was more effective than dabrafenib (Figure 1E). IF of KIM1 further supported this result (Figure 1F).

### 3.2 Cpd42 attenuated COM crystal-induced inflammation and necroptosis in HK2 cells

**FIGURE 2:**
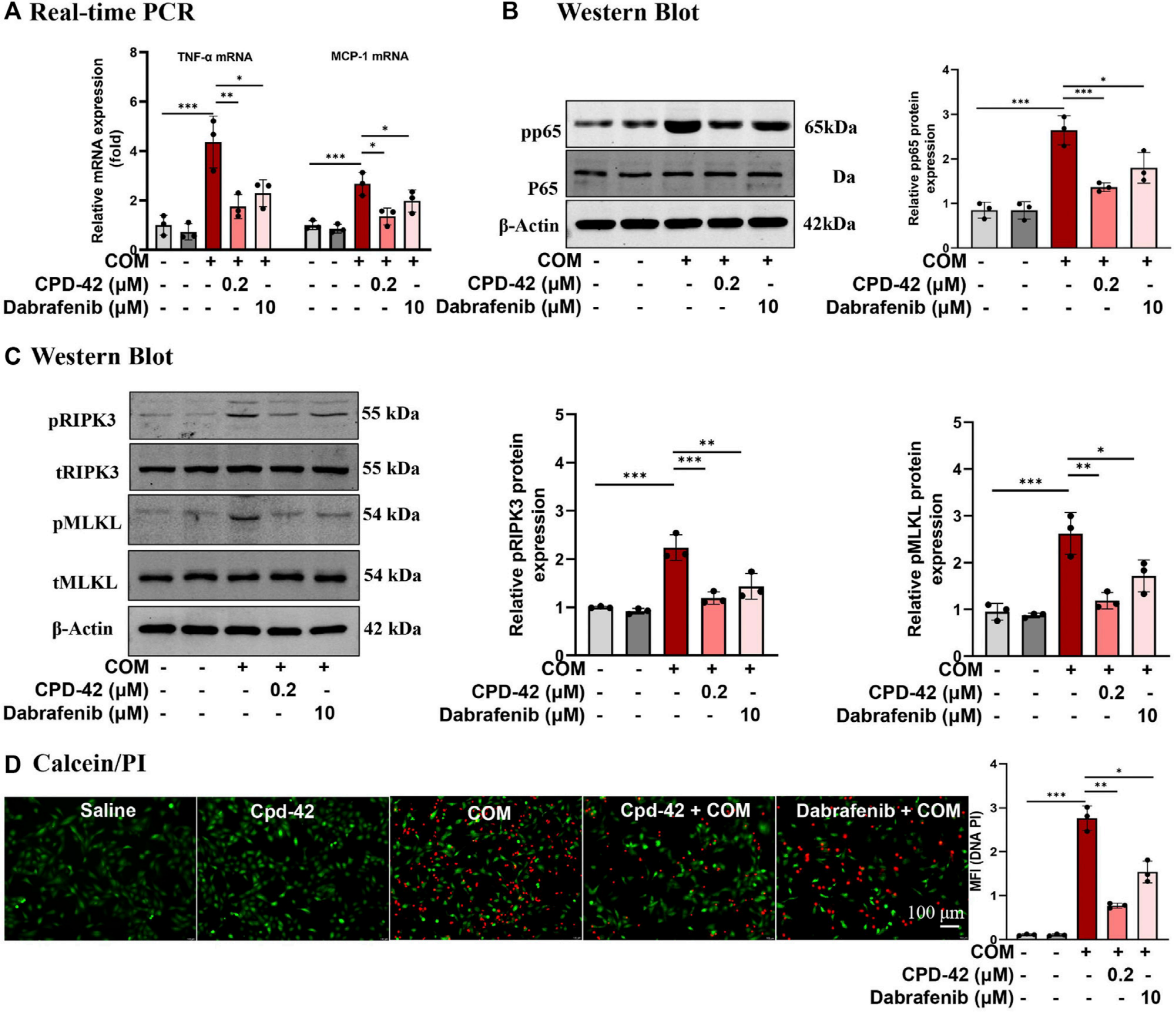
Compound 42 (Cpd-42) attenuates calcium oxalate monohydrate (COM) crystal-induced cell inflammation and necroptosis. **(A)** Real-time PCR analysis of TNF-α and IL-6. **(B)** Western blot analysis of p65 and phospho-p65. The results were determined by quantitative analysis of the greyscale values of the bands. **(C)** Protein expression of RIPK3, phospho-RIPK3, MLKL and phospho-MLKL was determined by Western blots, and the results were determined by quantitative analysis of the greyscale value of the bands. **(D)** Representative images of the calcein/Propidium Iodide (PI) assay in HK-2 cells, live cells with green fluorescence and dead cells with red fluorescence; Data are shown as the mean ± S.E.M. of 3 biological replicates. *p < 0.05, **p < 0.01, ***p < 0.001.

We stimulated HK2 cells with COM crystals, and RT‒PCR analyses demonstrated that the proinflammatory cytokines TNF-α and monocyte chemoattractant protein 1 (MCP-1) induced by COM crystals were significantly increased, while Cpd-42 (0.2 μM) significantly decreased the inflammatory response, and the anti-inflammatory effect of Cpd-42 was superior to that of dabrafenib (Figure 2A). Western blot analyses demonstrated that Cpd-42 significantly suppressed the phosphorylation of p65 induced by COM crystals (Figure 2B). Furthermore, Western blot analyses confirmed that Cpd-42 significantly inhibited the activation of the RIPK3-MLKL signalling pathway induced by COM crystals (Figure 2C). Calcein/PI staining also confirmed that Cpd-42 significantly reduced COM crystal-induced HK2 cell death (Figure 2D). More importantly, Cpd-42 (0.2 μM) inhibited inflammatory response, the phosphorylation of p65, and activation of the RIPK3-MLKL signalling pathway and HK2 cell necroptosis were superior to those of dabrafenib (10 μM) (Figure 2).

### 3.3 Cpd-42 protected against CaOx nephrocalcinosis-induced intrarenal inflammation and injury in mice

**FIGURE 3:**
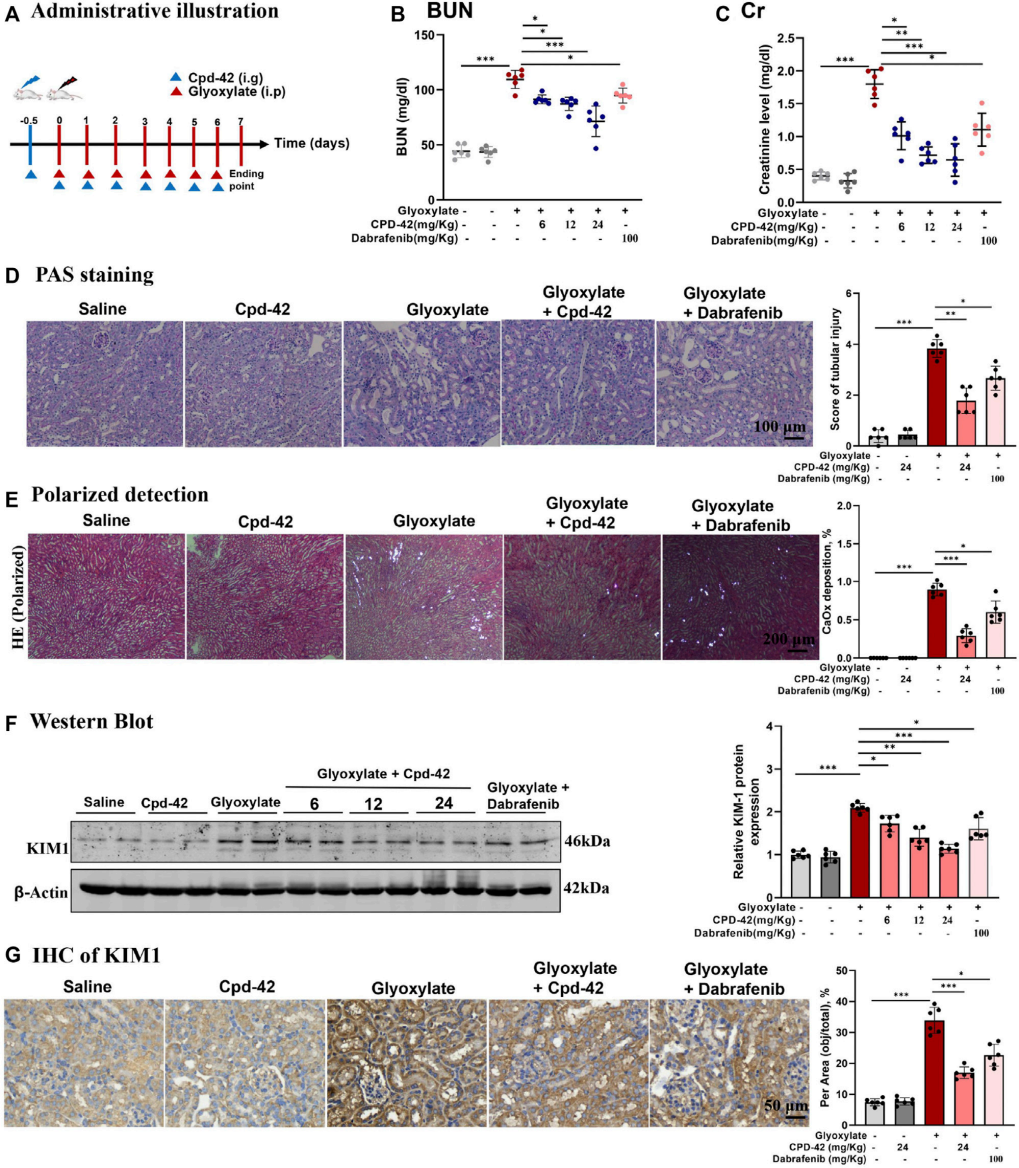
Compound 42 (Cpd-42) protected against Calcium oxalate (CaOx) crystal-induced renal injury in mice. **(A)** Illustration of Cpd-42 administered before and after induction of crystalline nephropathy with CaOx. ip = intraperitoneal, ig = intragastric. **(B,C)** Serum Cr and BUN in the mice with CaOx crystalline nephropathy with or without Cpd-42 administration. **(D)** PAS staining was used to illustrate and score tubular injury. **(E)** CaOx crystal deposition in the kidney was visualized by polarized light microscopy and quantified as the percentage of crystal deposition in each kidney section. **(F)** Western blot analysis of KIM1. The results were determined by quantitative analysis of the greyscale values of the bands. **(G)** Representative immunohistochemistry images and quantitative analysis of KIM1. Data are shown as the mean ± S.E.M. of 6 biological replicates. *p < 0.05, **p < 0.01, ***p < 0.001.

We next investigated the protective effect of Cpd-42 against CaOx nephrocalcinosis. Cpd-42 (6, 12 and 24 mg/kg) was administered by oral gavage to pretreated mice 12 h prior to glyoxalate injection, and dabrafenib (100 mg/kg) was used as a positive control (Figure 3A). The results showed that Cpd-42 markedly reduced the elevated serum Cr and BUN levels (Figures 3B,C) in the mouse model of CaOx nephrocalcinosis. PAS staining revealed that mice were subjected to severe pathological injury of renal tubular epithelial cells after CaOx exposure. Interestingly, renal tubular epithelial cell injury and CaOx crystal deposition in the kidney were also improved by Cpd-42 pretreatment (Figures 3D,E). Western blot and IHC results demonstrated that Cpd-42 markedly reduced KIM1 protein (Figures 3F,G). Notably, Cpd-42 at 24 mg/kg exhibited a superior protective effect against kidney injury to those of dabrafenib at 100 mg/kg (Figure 3).

**FIGURE 4:**
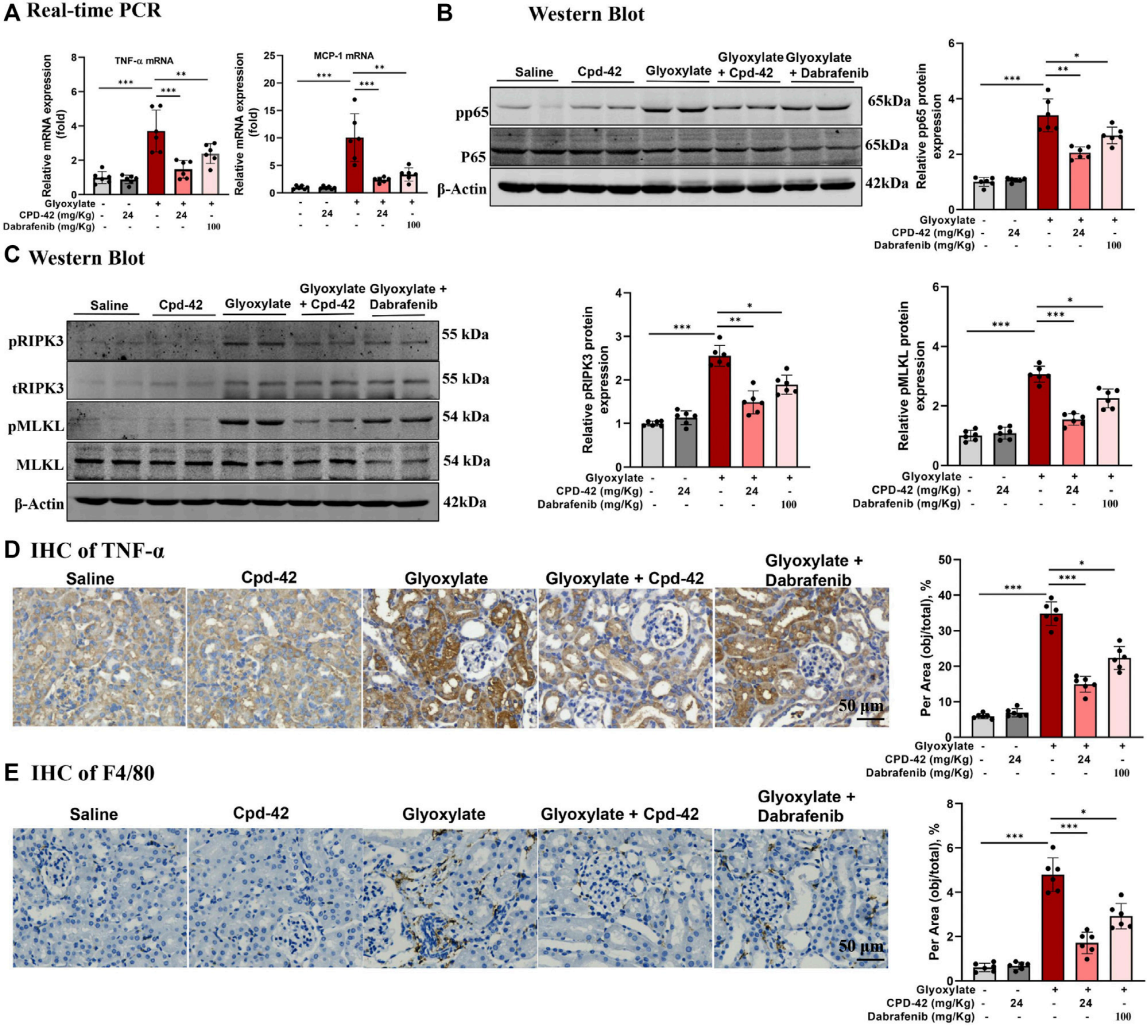
Compound 42 (Cpd-42) attenuates intrarenal inflammation and necroptosis in a mouse model of Calcium oxalate (CaOx) crystalline nephropathy. **(A)** Real-time PCR analysis of TNF-α and MCP-1. **(B,C)** Western blot and quantitative analysis of p65, phospho-p65, RIPK3, phospho-RIPK3, MLKL and phospho-MLKL. **(D,E)** Representative immunohistochemistry images and quantitative analysis of F4/80 and TNF-α. Data are shown as the mean ± S.E.M. of 6 biological replicates. *p < 0.05, **p < 0.01, ***p < 0.001.

Furthermore, we investigated the anti-inflammatory effects and inhibition of necroptosis of Cpd-42, and the results indicated that Cpd-42 reversed the mRNA expression of TNF-α and MCP-1 (Figure 4A), phosphorylation of p65 (Figure 4B) and activation of the RIPK3-MLKL signalling pathway (Figure 4C) in CaOx-induced nephropathy. Consistently, IHC of TNF-α (Figure 4D) and F4/80 (Figure 4E) also revealed that Cpd-42 reversed intrarenal inflammatory cytokine release and macrophage infiltration. In summary, Cpd-42 protected against CaOx nephrocalcinosis-induced intrarenal inflammation and injury in mice, and the protective effect of Cpd-42 was significantly better than that of dabrafenib.

### 3.4 Therapeutic effect of Cpd-42 in an established mouse model of CaOx nephrocalcinosis

**FIGURE 5:**
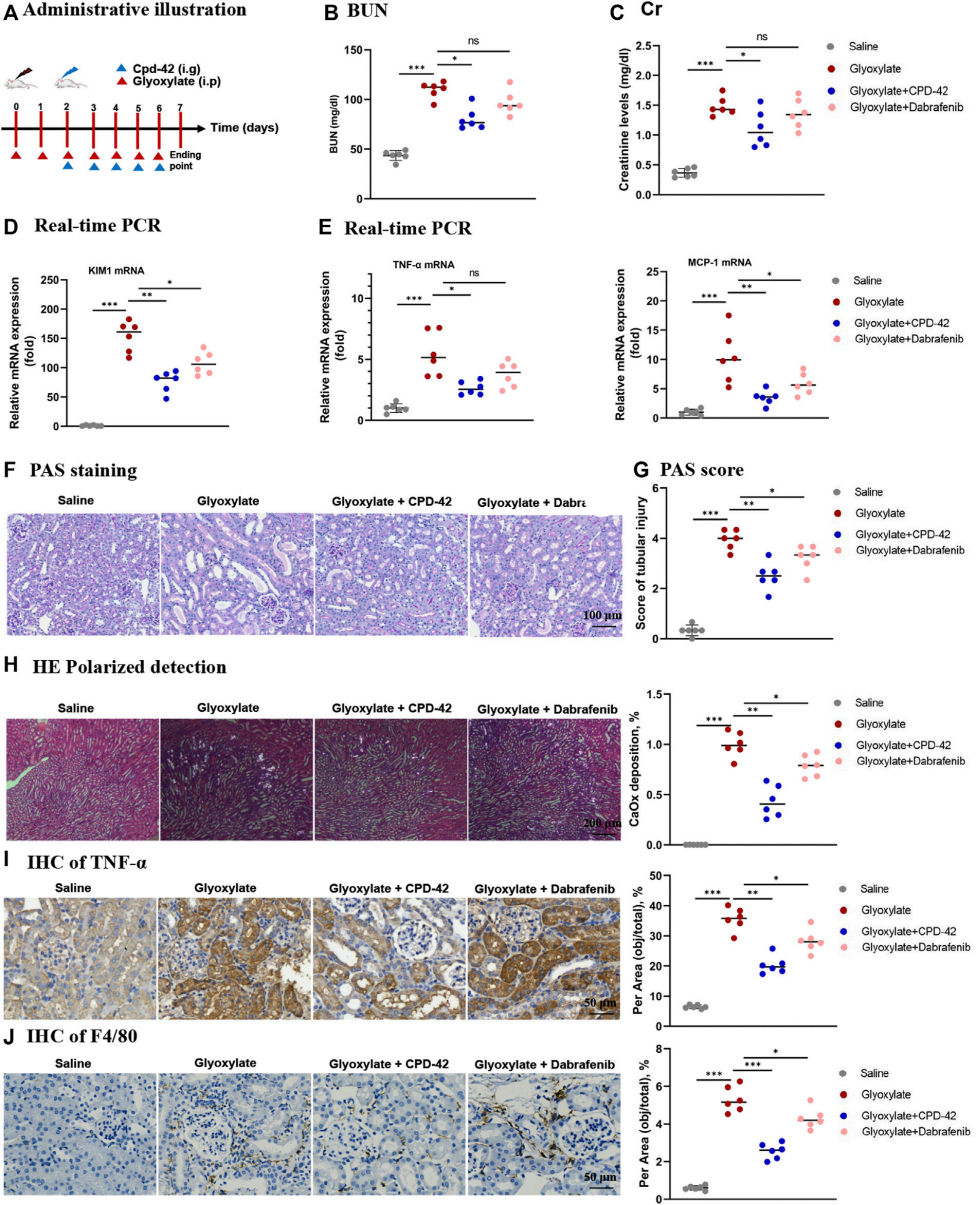
Compound 42 (Cpd-42) attenuates renal injury and inflammation in an established Calcium oxalate (CaOx)-induced nephropathy mouse model. **(A)** Illustration of Cpd-42 administered after the induction of crystalline nephropathy with CaOx. ip = intraperitoneal, ig = intragastric. **(B,C)** Serum Cr and BUN assay. **(D,E)** Real-time PCR analysis of KIM1, TNF-α and MCP-1. **(F,G)** PAS staining illustrated and scored tubular injury. **(H)** CaOx crystal deposition in the kidney and quantified as the percentage of crystal deposition in each kidney section. **(I,J)** Representative immunohistochemistry images and quantitative analysis of TNF-α and F4/80. Data are shown as the mean ± S.E.M. of 6 biological replicates. *p < 0.05, **p < 0.01, ***p < 0.001.

To explore the therapeutic effects of Cpd-42 in the CaOx nephrocalcinosis model, we administered Cpd-42 by gastrulation 3 days after beginning the glyoxalate injection (Figure 5A). The results showed that Cpd-42 treatment partially reversed the elevated BUN and Cr levels (Figures 5B,C), mRNA expression of KIM1, TNF-α and MCP-1 (Figures 5D,E) compared with glyoxalate group. PAS staining demonstrated that tubular injury was also partially reversed by treatment with Cpd-42 (Figure 5F and Figure G), while the deposition of CaOx crystals in the kidney was also reduced (Figure 5H). IHC also showed that Cpd-42 decreased TNF-α (Figure 5I) and macrophage infiltration (Figure 5J) in the CaOx-deposited kidneys. The results indicated that Cpd-42 has a favourable therapeutic effect on established CaOx nephrocalcinosis.

### 3.5 Cpd-42 exerted protective effects by specifically targeting and inhibiting RIPK3-mediated necroptosis

**FIGURE 6:**
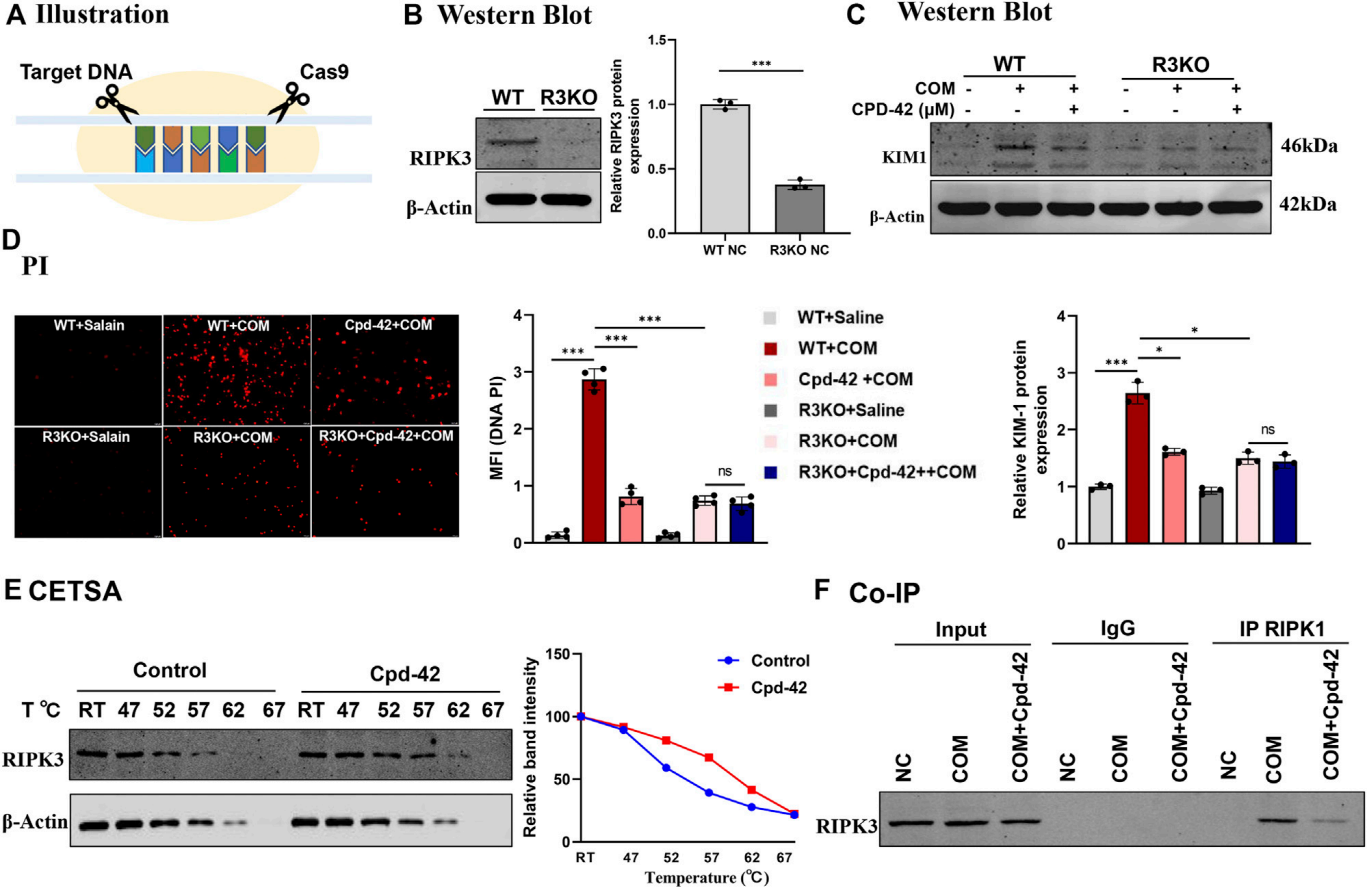
Compound 42 (Cpd-42) exerted renoprotective effects by directly binding to RIPK3. **(A)** Illustration of RIPK3 knockout (KO) cell generation by the CRISPR‒Cas9 system. **(B)** KO of RIPK3 was confirmed by Western blots. **(C,D)** Protein expression of KIM1 was determined by western blotting and Propidium Iodide (PI) staining in the RIPK3-KO cells exposed to calcium oxalate monohydrate (COM) crystals. Cpd-42 did not further attenuate cell injury and death when RIPK3 was knocked out. **(E)** Cellular thermal shift assay (CESTA) was used to assesses the stability of RIPK3 with or without Cpd-42 treatment. Cpd-42 enhanced the stability of RIPK3. **(F)** Coimmunoprecipitation with anti-RIPK1 detected the formation of RIPK1/RIPK3 necrosomes. Data are shown as the mean ± S.E.M. of at least 3 biological replicates. *p < 0.05, **p < 0.01, ***p < 0.001.

All the results above showed that Cpd-42 attenuated the cytotoxicity of CaOx crystals by inhibiting necroptosis *in vivo* and *in vitro*. To identify the specific mechanism by which Cpd-42 exerts protective effects, we generated RIPK3 knockout (KO) tubular epithelial cells using CRISPR/Cas system-mediated gene editing (Figure 6A). The RIPK3-KO cells were confirmed by Western blot analyses (Figure 6B). We then cocultured RIPK3-KO cells with COM crystals. Western blot analyses and PI staining indicated that RIPK3 KO significantly decreased KIM protein expression and cell death; however, RIPK3 KO cells pretreated with Cpd-42 did not show further enhancement of the protective effect of RIPK3 KO (Figures 6C,D). To further validate the interaction of RIPK3 proteins and Cpd-42, we conducted CETSA *in vitro*, which allowed us to assess target engagement. The results indicated that the thermal stability of RIPK3 was markedly elevated in the Cpd-42 treatment group when the denaturation temperatures ranged from 47°C to 67°C (Figure 6E). Furthermore, Co-IP showed that RIPK1 interacted with RIPK3 to mediate necroptosis induced by COM crystals, but Cpd-42 reduced formation of the RIPK1-RIPK3 necrosome (Figure 6F). All these results indicated that Cpd-42 was directly bound to the RIPK3 protein.

## 4 Discussion

Necroptosis has been shown to play an important role early in necroinflammation, and the blockade of necroptosis was able to prevent subsequent inflammatory and pathological injury mediated by immunity (Mulay et al., 2016; Lalaoui et al., 2020; Zheng and Kanneganti, 2020; Feoktistova et al., 2021; Yu et al., 2022). In this study, a specific and effective RIPK3 inhibitor, Cpd-42, was reported. *In vivo* and *in vitro*, Cpd-42 played a critical role in attenuating the cytotoxicity of CaOx crystals by inhibiting necroptosis and inflammation. Furthermore, Cpd-42 had a favourable therapeutic effect on established CaOx nephrocalcinosis model. More importantly, we confirmed that Cpd-42 exhibited superior inhibition of necroptosis and protection against renal tubular cell injury compared to dabrafenib.

Necroptosis is programmed necrosis triggered by the activation of RIPK1 and RIPK3. The combination of the two kinases forms RIPK1-RIPK3 necrosome in which RIPK1 and RIPK3 phosphorylate each other, leading to the activation of a downstream MLKL, which are key regulators of cell lysis and necrosis (Dannappel et al., 2014; Newton et al., 2014; Yuan et al., 2019; Samson et al., 2020). RIPK1, RIPK3 and MLKL are all targets of intervention against necroptosis-mediated inflammation and pathology (Dannappel et al., 2014; Mulay et al., 2016; Martens et al., 2020; Liu et al., 2022). To date, various inhibitors targeting RIPKs have been developed (Martens et al., 2020; Wang et al., 2021) and have been shown to protect against the progression of various kidney diseases (Mulay et al., 2016; Liu et al., 2018; Wang et al., 2019; Liu et al., 2022). We previously reported that two RIPK1 inhibitors, Cpd-71 and wogonin, attenuated cisplatin-induced nephropathy and were superior to the classical RIPK1 inhibitor Nec-1. However, necroptosis signals can be triggered by RIPK3 independently of RIPK1, which cannot be blocked by RIPK1, in addition RIPK1 has been identified to be involved in apoptotic and inflammatory signalling pathways (Moriwaki and Chan, 2016) and mutations in RIPK1 can lead to autoinflammatory diseases (Lalaoui et al., 2020). Dabrafenib was recently determined to be an inhibitor of RIPK3 kinase (Li et al., 2014) and was used to treat melanoma patients with the Braf^V600E^ gene mutation (Long et al., 2017); however, several studies showed that dabrafenib contributed to serious adverse events, such as hyperkeratosis, cutaneous squamous cell carcinoma and keratoacanthoma, arthralgia and haematological abnormalities (Hauschild et al., 2012; Dummer et al., 2020). The multitargeted nature of dabrafenib may be accompanied by serious additional adverse events in the treatment of necroptosis-associated CaOx nephrocalcinosis. Therefore, the identification of specific novel inhibitors for necroptosis with lower cytotoxicity is of clinical interest.

The novel RIPK3 inhibitor Cpd-42 exerted protective effects by specifically targeting RIPK3-mediated necroptosis. Previous studies by Zhang H et al. have demonstrated that Cpd-42 was over 60-fold more selective for RIPK3 than RIPK1 with low cytotoxicity (Zhang et al., 2019). We assessed the protective effects of Cpd-42 against CaOx nephrocalcinosis and found that this molecule demonstrated excellent affinity for RIPK3. We first showed that Cpd-42 markedly suppressed the activation of the RIPK3-MLKL signalling pathway by blocking the formation of the RIPK1-RIPK3 necrosome to attenuate kidney injury and reduce CaOx crystal deposition in the kidney. Furthermore, in the COM-stimulated RIPK3-KO cells, pretreatment with Cpd-42 failed to further exert protective effects, which indicated that RIPK3 was the critical point at which Cpd-42 exerts its physiological effects. CETSA also further confirmed the direct binding of Cpd-42 to RIPK3. Notably, Cpd-42 exhibited a superior anti-necroptosis effect compared to dabrafenib.

Increasing evidence has demonstrated that activation of the NLRP3-NF-κB signalling pathway and subsequent release of inflammatory cytokines are major factors in renal inflammation induced by CaOx nephrocalcinosis (Anders et al., 2018; Liu et al., 2019; Shee and Stoller, 2022). Necroptosis triggers an inflammatory response in various diseases (Wang et al., 2020; Zheng and Kanneganti, 2020). In a previous study by Welz et al. (Welz et al., 2011), RIPK3 deficiency protected against cell inflammation and death in spontaneous colitis and ileitis models induced by FADD deficiency, which indicates that necroptosis of cells regulated by RIPK3 can lead to intestinal inflammation. A recent study also indicated that NF-κB-mediated inflammation regulated by RIPK3 plays an essential role in a *β* cell dysfunction model and that RIPK3 inhibition can suppress the inflammatory process (Yang et al., 2020). In the present work, Cpd-42, by blocking RIPK3-mediated necroptosis, attenuated the activation of NF-κB signalling, macrophage infiltration and the release of inflammatory cytokines. Moreover, Cpd-42 has been confirmed to exhibit a stronger anti-inflammatory effect than dabrafenib.

In conclusion, we synthesized and characterized a novel RIPK3-targeting inhibitor, Cpd-42, which exhibited excellent anti-necroptosis and anti-inflammatory effects to attenuate the cytotoxicity of CaOx crystals and reduce renal CaOx crystal deposition induced by glyoxylate. We also described a favorable therapeutic effect of Cpd-42 on established CaOx nephrocalcinosis model. Although these findings need to be further investigated, targeted inhibition of RIPK3-mediated necroptosis with Cpd-42 may provide a promising therapeutic approach for clinical CaOx nephrocalcinosis.

## Data Availability

The original contributions presented in the study are included in the article/Supplementary Material, further inquiries can be directed to the corresponding authors.

## Supplementary material

Supplementary Figure S1 The chemical structure of Compound 42 (Cpd-42) was confirmed by mass spectrometry. Cpd-42：[M+H]+:C24H19BrFN4O3S+：Calculated m/z: 541.0340 (100.0%), 543.0320 (97.3%), 542.0374 (26.0%), 544.0353 (25.3%). Found：541.0344,542.0371,543.0329,544.0352.
