# Supplementary figures and images for: Cpd-42 protects against calcium oxalate nephrocalcinosis-induced renal injury and inflammation by targeting RIPK3-mediated necroptosis
Source: Front Pharmacol. 2022 Nov 3;13:1041117. doi: 10.3389/fphar.2022.1041117 (PMC9669592; doi:10.3389/fphar.2022.1041117)

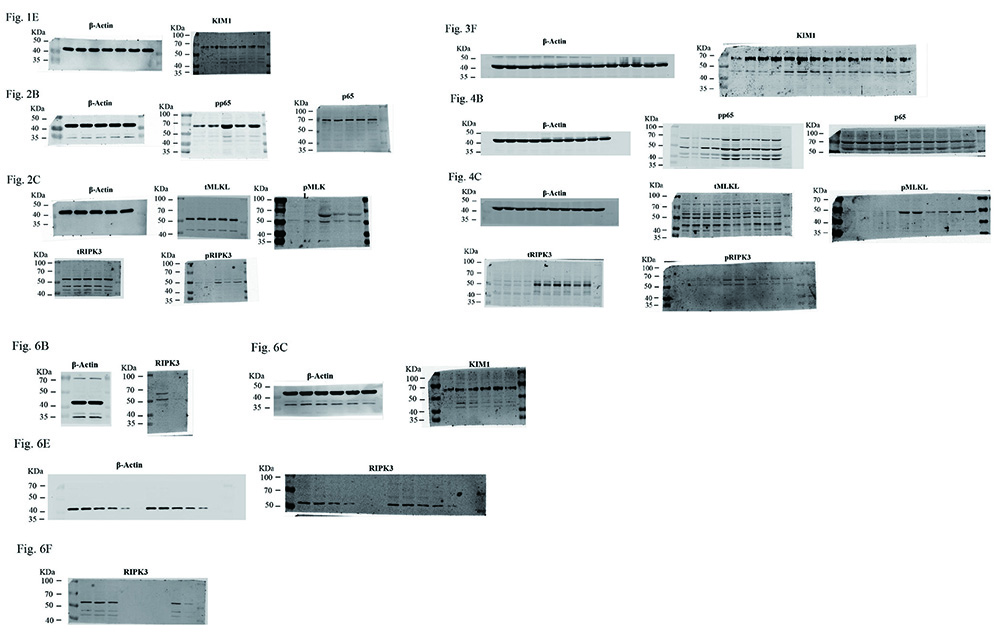

Supplement: Supplementary file 1 [file Image1.JPEG]

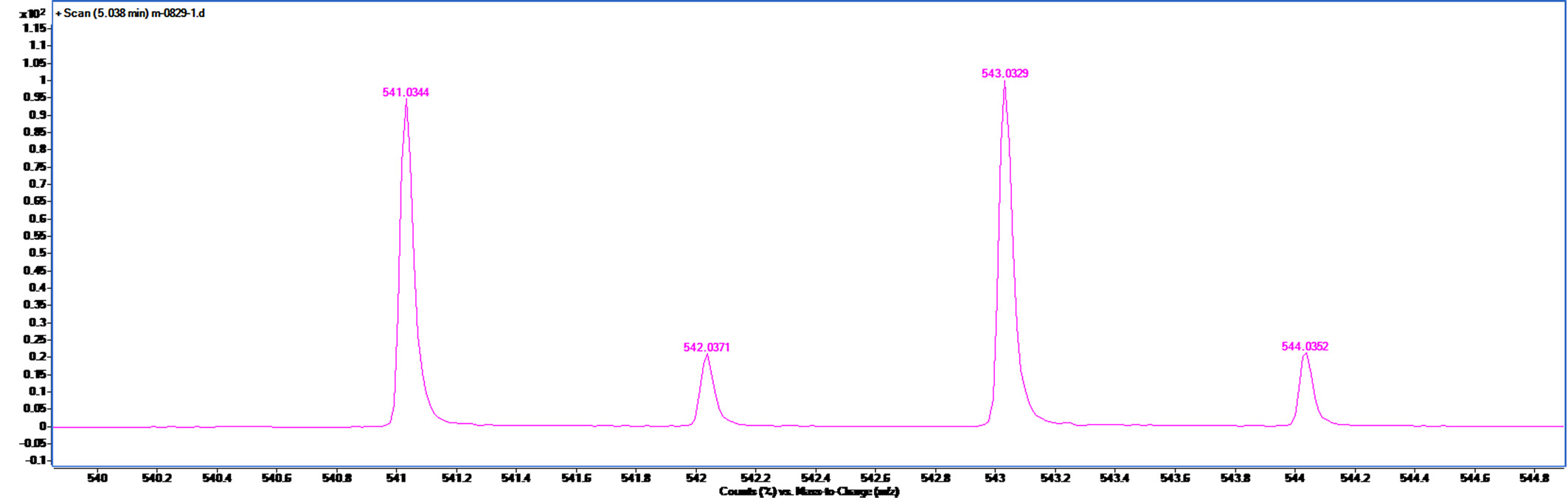

Supplement: Supplementary file 2 [file Image2.JPEG]
